# Editorial: Bone and Cartilage Regeneration With Extracellular Vesicles

**DOI:** 10.3389/fbioe.2021.692836

**Published:** 2021-05-24

**Authors:** Roberta Tasso, Susanne Grässel, Frank Zaucke

**Affiliations:** ^1^Department of Experimental Medicine (DIMES), University of Genova, Genoa, Italy; ^2^Department of Orthopaedic Surgery, Experimental Orthopaedics, Centre for Medical Biotechnology (ZMB/Biopark 1), University of Regensburg, Regensburg, Germany; ^3^Dr. Rolf M. Schwiete Research Unit for Osteoarthritis, Department of Orthopedics (Friedrichsheim), University Hospital Frankfurt, Goethe University, Frankfurt, Germany

**Keywords:** extracellular vesicles, exosomes, microvesicles, musculoskeletal repair, regenerative medicine, tissue engineering

The primary aim of regenerative medicine is to stimulate tissue healing. Despite the great promises raised by the use of stem/progenitor cells for skeletal tissue regeneration, cell tracking analysis has revealed that transplanted cells do not commonly become part of the injured tissue (Pittenger et al., 2019). The role of the stem cell-derived secretome is becoming increasingly intriguing due to its ability to stimulate endogenous regenerative processes. Therefore, the so-called “paracrine hypothesis” has taken hold (Gnecchi et al., 2016). Extracellular vesicles (EVs) are important components of the cell secretome, representing promising tools for the delivery of biologically active molecules which can be used for therapeutic purposes (Alcaraz et al., 2019). EVs are bilayer membrane fragments released by almost any cell type upon activation or death. Two major types of EVs are usually distinguished: exosomes, formed from the endosomal cell compartment, and microvesicles, produced by the direct extrusion from the cell plasma membrane (Doyle and Wang, 2019). Nowadays, the effects specifically exerted by one EV subpopulation over another are still unclear. This is in part due to their overlapping size and variable cargos, which does not allow a precise discrimination between them. EVs contain proteins, lipids, and nucleic acids and have the potential to activate not only complementary, pro-regenerative signaling pathways in the same responder cells, but also to stimulate multiple target cell populations and tissues. This property could make them an efficient therapeutic vehicle for bone and cartilage regenerative medicine. However, there are many concerns that should be addressed, such as the development of strategies to obtain sufficient amounts of EVs, the identification and characterization of the optimal donor cell source, the necessity to develop ideal scaffolds to be used as depots for controlled release of EVs, as well as the need to better understand the mechanisms underlying bone/cartilage formation after EV treatment.

This Research Topic deals with these and other key issues in the field of bone and cartilage regeneration through the use of EVs. For instance, Oliveira et al. evaluated the impact of milk-derived EVs (mEVs) on the dynamics of bone loss in mice. In this article, mEVs were supplemented in the drinking water of mice that were either developing diet-induced obesity or receiving ovariectomy (OVX). The authors demonstrated that diet-induced obese mice treated with mEVs were protected from bone loss, while the number of osteoclasts in the femur of OVX mice and the osteoclastogenic potential of their bone marrow progenitors were significantly lowered upon mEV supplementation. Interestingly, the RANKL/OPG ratio increased systemically and locally in both models and was rescued by mEV treatment.

Another interesting study by Otahal et al. explored the possibility of applying EVs present in the platelet rich plasma (PRP) and in the hyperacute serum (HypACT) in osteoarthritis (OA). OA-patient derived chondrocytes were treated with either PRP-EVs or hypACT-EVs, and their effects were compared to the corresponding total blood products. The authors reported that both EV preparations were able to elicit chondroprotective expression of anabolic genes and to reduce the expression of inflammatory cytokines, suggesting that blood-derived EVs could be attractive regulators of cartilage extracellular matrix metabolism and inflammation.

The possibility of using Bone Marrow-Mesenchymal Stromal Cell (BMSC)-derived EVs as therapeutic tools for the treatment of OA related osteoporosis (OP) has been proposed by Niedermair et al. Assuming that EVs isolated from bone cells are able to modulate (patho)physiologic processes related to bone remodeling and repair, the authors investigated whether EVs derived from osteoblasts of patients with hip OA [coxarthrosis (CA)], OP, or a combination of both (CA/OP), were able to affect the metabolism and osteogenic differentiation capacity of BMSCs. Stimulation of BMSCs with EVs isolated from CA, OP, and CA/OP osteoblasts had mostly catabolic effects on cell metabolism and osteogenic differentiation irrespective of donor pathology, reflecting the critical impact of tissue microenvironment on cell metabolism. In another study from the same group, Li et al. investigated *in vitro* the potential protective effects of BMSC-derived EVs on IL-1β stimulated chondrocytes obtained from OA-patients. BMSC derived EVs promoted proliferation and attenuated IL-1β-induced reduction of chondrocyte migration whereas inhibiting apoptosis of responder chondrocytes via down-regulation of pro-inflammatory signaling pathways (Erk1/2, PI3K/Akt, p38, TAK1, and NF-kB). These effects were not dependent on IL-1β stimulation. Finally, two reviews summarized current knowledge on the role of EVs in musculoskeletal regeneration. Herrmann et al. discussed the role of EVs in musculoskeletal research and summarized the mechanisms by which EVs contribute to musculoskeletal tissue homeostasis, regeneration and disease. In particular, the authors focused on MSCs, being the most frequently used cell source for EV generation for musculoskeletal applications. They reviewed the EV-mediated matrix remodeling and mineralization process, pro-angiogenic effects and immunomodulatory activities in bone healing, OA, OP, and trauma. This review article also summarizes important practical considerations for EV-based therapies like EV isolation and enrichment protocols. Velot et al. focused their mini review on the promotion of cartilage regeneration by EVs isolated from various cell sources (mature or stem cells among others), and on the beneficial effects of cargoes produced from natural or tuned EVs.

The issues discussed in this Research Topic address several important biological aspects related to the use of EVs for bone and cartilage repair. Despite the great amount of work already performed, a more detailed molecular profiling of the vesicle cargo, as well as a more stringent EV characterization will allow to further define novel cell-free based therapeutic and tissue engineering strategies for the treatment of bone and cartilage-related pathologies.

## Author Contributions

All authors listed have made a substantial, direct and intellectual contribution to the work, and approved it for publication.

## Conflict of Interest

The authors declare that the research was conducted in the absence of any commercial or financial relationships that could be construed as a potential conflict of interest.
